# Production and use of dry-rolled hybrid rye grain as a replacement for barley grain on growth performance and carcass quality of feedlot steers

**DOI:** 10.1093/tas/txae059

**Published:** 2024-04-11

**Authors:** Fuquan Zhang, Rachel E Carey, Rebecca S Brattain, Herman Wehrle, Gregory B Penner

**Affiliations:** Department of Animal and Poultry Science, University of Saskatchewan, Saskatoon, Saskatchewan, CanadaS7N 5A8; Department of Animal and Poultry Science, University of Saskatchewan, Saskatoon, Saskatchewan, CanadaS7N 5A8; KWS Cereals USA, LLC, Champaign, IL 61822, USA; KWS Seeds Canada, Calgary, AB, CanadaT2T 0A4; Department of Animal and Poultry Science, University of Saskatchewan, Saskatoon, Saskatchewan, CanadaS7N 5A8

**Keywords:** cereal grain, liver abscesses, starch

## Abstract

The objectives were to compare cereal grain and straw yield between barley and hybrid rye (**HR**) and to evaluate whether the inclusion of dry-rolled HR grain as a replacement for barley grain affected feed intake and growth for growing cattle, and feed intake, growth, and carcass characteristics for finishing cattle. Crop yield was measured by directly weighing harvested grain and straw bales (*n* = 3 plots/grain type). Three-hundred sixty steers with an initial body weight (**BW**) of 348 ± 40 kg were stratified by BW and randomly assigned to 1 of the 24 pens during the growing phase (*n* = 8; 65 d). The control diet (**BCON**) included 60.22% barley grain with HR included by replacing 50 (**BMID**) or 100% (**BHIGH**) of the barley grain on a dry matter (**DM**) basis. Steers were re-randomized for the finishing phase (*n* = 6; 118 d) and treatments included a control diet containing 88.60% barley grain (**FCON**) with HR replacing 33 (**FLOW**), 67 (**FMED**), or 100% (**FHIGH**) of the barley grain (DM basis). The grain yield was greater (*P* = 0.04) and straw yield tended (*P* = 0.06) to be less for HR than barley. There were no effects of HR inclusion on DM intake (DMI) or G:F during the growing phase, but average daily gain (ADG) responded quadratically (*P* = 0.02) with cattle fed 50% HR having the greatest gain. During finishing, DMI decreased linearly as HR grain inclusion increased (*P* < 0.01). ADG initially increased from FCON to FLOW followed by a decrease with increasing HR inclusion (quadratic, *P* < 0.01), but G:F was not affected. Hot carcass weight was greatest for FCON with the magnitude of difference between FCON and the HR treatments increasing with increasing inclusion of HR (quadratic, *P* = 0.02). There was a linear increase in dressing percentage (*P* = 0.02) and a linear reduction in back fat thickness (*P* = 0.04) with increasing inclusion of HR. Increasing the inclusion of HR during finishing cubically (*P* < 0.01) affected the proportion of minor and severe liver abscesses with an average of 34.60% severely abscessed livers when HR was included compared to 11.11% for BCON. HR may have greater grain yield than barley, and partial replacement of barley grain with HR may improve ADG without affecting DMI or G:F during the growing phase. However, replacing barley grain in finishing diets with HR decreases DMI, and increases the risk of minor and severe liver abscesses, but does not affect feed conversion, suggesting HR should not replace more than 33% of the barley grain to maintain ADG.

## Teaser

This study demonstrated that hybrid rye has greater grain yield than barley grain and that feeding hybrid rye as a replacement for barley grain may improve average daily gain during the growing phase. Increasing inclusion of hybrid rye as a replacement for barley grain linearly reduced dry matter intake and increased liver abscess.

## Introduction

Fall rye is a winter cereal grown in western Canada and the upper Midwestern United States (McGhee and Stein, 2020) due to adequate winter survival, and tolerance to drought and poor soil conditions (Geiger and Miedaner, 2009). The vast majority of rye (50% to 75%) is harvested for baking, with the remainder used in the distilled spirits industry, and for biofuel production (Geiger and Miedaner, 2009; Arendt and Zannini, 2013). Rye has not traditionally been included in feedlot diets because of concerns over ergot alkaloid contamination (Coufal-Majewski et al., 2016; Bederska-Łojewska et al., 2017; European Food Safety Authority et al., 2017). However, risk for ergot alkaloid contamination with new hybrid rye (**HR**) varieties containing the Rfp1 gene (KWS, Lochow, Germany) has been reduced (Hackauf et al., 2012; Miedaner et al., 2017) providing new opportunities for the use of HR in diets for feedlot cattle.

The starch and crude protein (**CP**) concentrations for rye are similar to barley grain (NASEM 2016). However, the in situ degradation rate and effective degradability of starch are reported to be greater for rye (116.5%/h and 96.2% of starch) than barley (85.1%/h and 95.0% of starch; Krieg et al., 2017). Therefore, the risk for and severity of low ruminal pH may increase when feeding high proportions of rye grain in finishing cattle diets (Krieg et al., 2017; Rajtar et al., 2021; Rusche, 2021). That said, available data describing performance and secondary indicators of ruminal acidosis for cattle-fed HR are limited (Rusche et al., 2020). Moreover, there are currently no published studies that compare barley and HR for growing and finishing beef cattle.

The hypotheses of the current study were that: (1) cereal grain production of fall-seeded HR would be greater than that of spring-seeded barley grain; (2) replacing barley grain with HR would not affect feed intake or growth for growing steers; and (3) increasing the inclusion of dry-rolled HR grain, at the expense of dry-rolled barley grain, would decrease dry matter (**DM**) intake (DMI) and growth performance, and increase the frequency of liver abscesses when fed to finishing cattle. Thus, the objectives of the study were to evaluate: (1) cereal grain and straw production for HR and barley grain; (2) DMI and growth during the growing phase when provided diets with increasing inclusion of HR as a replacement for barley grain; and (3) DMI, growth, and carcass characteristics for finishing cattle fed diets containing increasing inclusion rates of dry-rolled HR grain as a replacement for dry-rolled barley grain.

## Materials and Methods

All use of cattle in this project was approved by the University of Saskatchewan (Saskatoon, Saskatchewan, Canada) Animal Research Ethics Board (protocol 20210069) prior to the research being conducted. In addition, animal husbandry followed the guidelines provided by the Canadian Council on Animal Care for Farm Animals (Ottawa, ON, Canada).

### Study 1: Crop Production

HR grain (seeded at 73.08 kg/ha on September 18th and 19th, 2019; *Secale cereale L*.; KWS Bono, FP Genetics, Regina, SK, Canada) and barley grain (seeded at 107.60 kg/ha on May 13th and 14th, 2020; *Hordeum vulgare L*.; CDC Austenson, SeCan, Kanata, ON, Canada) were randomly assigned to 1 of 6 plots (3 plots/grain type; 13.2 ± 1.85 ha/plot) at the University of Saskatchewan’s Livestock and Forage Center of Excellence (Clavet, SK, Canada). Both crops were seeded on Orthic Dark Brown Chernozemic soil formed in loamy lacustrine materials without irrigation (Bradwell, SK, Canada; SKSIS Working Group, 2018). The HR plots were sprayed 1 to 2 d before seeding with glyphosate for weed suppression (2.47 L/ha; Stonewall 540; WinField United Canada, Saskatoon, SK, Canada). Barley plots were sprayed 7 to 8 d before seeding with florasulam (Priority; Adama Agricultural Solutions Canada Ltd., Winnipeg, MB, Canada) and glyphosate (Transorb HC; Bayer Cropscience Inc., Calgary, AB, Canada) at 0.26 and 0.82 L/ha, respectively. For HR, fields were fertilized with granular fertilizer to provide 97 kg/ha N, 36 kg/ha P, and 8.5 kg/ha K and for barley, granular fertilizer added 12 kg/ha N, 31 kg/ha P, 10 kg/ha K, and 14 kg/ha S. Anhydrous ammonia was applied to barley grain plots between May 1 and 3, 2020 providing an additional 27 kg of N/ha. All fertilizer applications followed recommendations from a local agronomist and were specific to the crop grown. HR grain was swathed on August 11th and 12th, 2020 and then harvested using a John Deere 9610 combine (Deere and Co., Moline, IL, USA) on August 18th and 19th, 2020. Barley grain was harvested using the same combine fit with a direct cut header on August 19th and 20th, 2020. Total precipitation during the fall, winter, spring, and summer (September 2019 to August 2020) equated to 68% (45 mm), 82% (18 mm), 83% (63 mm), and 101% (175 mm) of the 10-yr average (Environment and Climate Change Canada, 2024).

The grain yield from each plot was recorded by weighing each load of the harvested grain on a calibrated scale. Grain density was determined using a Canadian Cox Funnel and a 500-mL standard metric cup (Seedburo Equipment Company, Des Plaines, IL, USA). In addition, sub-samples of grain collected from each truckload were composited for each grain source within a plot for further analysis. The composite samples were dried in a forced-air oven at 55°C until reaching a constant weight for DM determination. Subsequently, samples were ground using a hammer mill (Christie & Norris Lab Mill, Christie Turner Ltd., Ipswich, UK) to pass through a 1-mm sieve and were sent to Cumberland Valley Analytical Services (Waynesboro, PA, USA) for analysis of CP, starch, and ether extract as described by (Delver et al., 2023). Additionally, neutral detergent fiber (**NDF**) concentration was determined according to the method of Van Soest et al. (1991) utilizing sodium sulfite and α-amylase without ash correction. Calcium and P concentrations were determined using method 985.01 (AOAC 2000). Grain samples were also sent to Prairie Diagnostic Services Inc. (Saskatoon, SK, Canada) for ergot alkaloid analysis as described by Grusie et al. (2017). The HR grain had an ergot alkaloid concentration that exceeded the maximum tolerances allowed by the Canadian Food Inspection Agency (Ottawa, ON, Canada) with an average ± SD of 5.33 ± 2.51 mg/kg. The primary alkaloids were ergocristine/inine (2.26 ± 0.87 mg/kg), ergocryptine/inine (0.95 ± 0.55 mg/kg), ergotamine/inine (0.78 ± 0.37 mg/kg), ergocornine/inine (0.66 ± 0.35 mg/kg), ergometrine/inine (0.42 ± 0.24 mg/kg), and ergosine/inine (0.27 ± 0.15 mg/kg). As such, HR grain was cleaned using a wind and gravimetric screening approach with 2-mm sieves followed by color sorting to remove ergot bodies. Analysis of ergot alkaloid concentrations after cleaning confirmed that the HR grain fell within the Canadian Food Inspection Agency tolerances with mean values for total ergot alkaloids of 0.29 ± 0.09 mg/kg. The total ergot alkaloid concentration from the composited barley samples (*n* = 6) was ≤ 0.027 mg/kg on a DM basis with only 3 of the 6 barley samples having detectable values.

Additionally, the straw from each plot was baled using a John Deere 596 baler (Deere and Co.). At the time of baling, the total number of bales were recorded and a random subset of the bales (20% of the total bales from each plot) were weighed on a calibrated scale for determination of straw yield. Straw samples were obtained by core-sampling the weighed bales and samples were dried in a forced-air oven (55 °C) until achieving a constant weight. Samples were then ground for chemical analysis of DM, CP, acid detergent fiber (ADF), NDF, ash-free NDF (aNDFom), starch, ether extract, lignin, Ca, and P according to the same protocol as described above and by Delver et al. (2023).

### Study 2: Effect of Replacing Barley Grain With HR Grain for Growing Steers

Study 2 was conducted from February 16 to April 20th, 2022 at the Livestock and Forage Center of Excellence Beef Cattle Research and Teaching Unit (Clavet, SK, Canada). Three hundred and sixty steers were sourced from a commercial auction market and fed a common diet (barley silage 56.5%, barley grain 17.0%, oat hulls 20.0%, canola meal 6.0%, limestone 0.5% (as fed basis), with minerals and vitamins added using a micro-machine (Micro-Weigh System; Micro Technologies Feedlot Solutions, Amarillo, TX, USA)) until reaching a mean body weight (**BW**) of 348 ± 40 kg prior to the start of the study. Upon arrival to the feedlot, steers were given a management tag, and vaccinated against infectious bovine rhinotracheitis, bovine viral diarrhea, parainfluenza type 3, bovine respiratory syncytial virus, clostridial diseases, and histophilosis (Bovi Shield Gold One Shot and Ultrabac 7/Somubac; Zoetis Canada, Kirkland, QC, Canada) and topically treated for internal and external parasites (Bovimectin Pour-on; Bimeda Canada, Cambridge, ON, Canada). Steers were also implanted upon arrival with Ralgro (36 mg zeranol; Merck Animal Health, Kirkland, QC, Canada).

Steers were housed in soil-surfaced pens measuring 12 × 24 m with a 3.3-m high windbreak fence (15% porosity) at the back of each pen blocking prevailing winds from the north-northwest. The pens were fitted with fence-line concrete bunks providing 80 cm of linear bunk for each steer. An automatic water bowl, shared between two adjacent pens provided water ad libitum. All pens were bedded with HR straw at the same time throughout the study as needed.

Steers were blocked by BW and randomly assigned to 1 of the 24 pens (15 steers/pen). Pens, within a block, were then randomly assigned to 1 of the 3 dietary treatments (Table 1) dispersed throughout the feedlot. All growing diets included 60.22% grain (DM basis) with HR replacing 0% (**BCON**), 50% (**BMED**), or 100% (**BHIGH**) of the barley grain in diets for growing steers. Urea was added to balance CP among diets given the rapidly degradable CP concentrations in both barley and rye grain (Krieg et al., 2018) and to avoid confounding effects caused by dietary CP. Steers were weighed on two consecutive days at the start and end of the growing phase to determine initial and final BW. The average of the 2-d measurements were used to determine the starting and ending BW. The BW measurements were initiated at 0830 hours (prior to feeding) to reduce the effect of gut fill on BW. In addition, steers were weighed every 3 weeks on a single day with BW data used to calculate average daily gain (**ADG**) by regressing unshrunk BW with day of the study. The gain-to-feed (**G:F**) ratio was calculated by dividing the unshrunk ADG by the average DMI. Methods to calculate DMI are described in the grain processing and feeding management section below.

**Table 1.** Ingredients and chemical composition of diets used during the growing period

	Hybrid rye inclusion rate^*^		
Item	BCON	BMED	BHIGH
Ingredients, % of DM			
Corn silage	7.04	7.04	7.04
Barley silage	27.95	27.95	27.95
Oat hulls	3.44	3.00	2.54
Barley grain (CDC Austenson)	60.22	30.11	—
Hybrid rye grain (KWS Bono)	—	30.11	60.22
Limestone	1.25	1.25	1.25
Urea	—	0.44	0.90
Mineral^†^	0.10	0.10	0.10
Chemical composition, % DM^‡^			
DM	69.99 ± 0.24	70.89 ± 0.28	71.78 ± 0.33
OM	93.62 ± 0.26	93.45 ± 0.30	93.26 ± 0.34
CP	16.13 ± 0.35	16.37 ± 0.46	16.66 ± 0.57
ADF	16.13 ± 0.52	14.35 ± 0.38	12.56 ± 0.24
NDF	34.26 ± 1.00	32.84 ± 0.62	31.40 ± 0.41
Starch	33.08 ± 1.47	35.18 ± 1.36	37.28 ± 1.31
Ether extract	3.26 ± 0.004	3.02 ± 0.07	2.78 ± 0.14
Ca	0.62 ± 0.002	0.65 ± 0.05	0.68 ± 0.11
P	0.33 ± 0.006	0.30 ± 0.002	0.26 ± 0.003

^*^The treatment diets contained hybrid rye grain at 0%, 30.11%, or 60.22% of dietary DM for BCON, BMED, and BHIGH, respectively.

^†^Mineral contained: Ca: 4.00 %; fat: 1.00%; Mn: 120,000 mg/kg; Cu: 60,000 mg/kg; Zn: 180,000 mg/kg; I: 5,000 mg/kg; Co: 750 mg/kg; Se: 750 mg/kg; vitamin A: 25,200 IU; vitamin D: 2,520 IU; vitamin E: 158 IU; 33 mg/kg of monensin (Elanco Animal Health, Greenfield IN) on a DM basis.

^‡^Chemical composition is expressed as means ± SD (*n* = 3).

On day 45 of the growing phase, 24 samples of freshly defecated feces (250 g/sample) were collected from at least 6 steers/pen. Samples were dried in a forced-air oven at 55 °C for 96 h for DM determination. Dried fecal samples were then ground using a hammer mill to pass through a 1-mm screen. The ground samples were analyzed for determination of fecal starch using near-infrared spectroscopy with previously validated calibrations (Jancewicz et al., 2017).

### Study 3: Effect of Replacing Barley Grain With HR Grain for Finishing Steers

Following completion of Study 2, steers were re-implanted with 120 mg of trenbolone acetate and 24 mg of estradiol (Revalor-S; Merck Animal Health) and used in a finishing study (April 21st to August 5th, 2022). Steers were blocked by BW and the dietary treatments were applied during the growing phase into 1 of the 24 pens were then randomized to dietary treatments (6 pens/treatment) dispersed throughout the feedlot. Treatments included HR at the expense of barley grain (Table 2) with inclusion rates of: 0 (**FCON**); 33 (**FLOW**); 67 (**FMED**); and 100% (**FHIGH**). Given the diets all contained 88.60% cereal grain, the treatment structure imposed allowed for final finishing diet HR grain inclusion rates of 0.00%, 29.24%, 59.36%, and 88.60% (DM basis). The diets were formulated to supply sufficient energy and protein for finishing cattle gaining 2.0 kg/d, along with minerals and vitamins to meet or exceed requirements (NASEM, 2016). No attempt was made to make diets isonitrogenous as the predicted supply of metabolizable protein exceeded requirements. The transition to the high-grain diet (20 d) was included in the performance results as treatment diets were initiated at the start of the dietary transition (Supplementary Table 1).

**Table 2.** Ingredients and chemical composition of diets used during the finishing period

Item	Hybrid rye inclusion rate^*^			
	FCON	FLOW	FMED	FHIGH
Ingredients, % of DM				
Barley silage	10.00	10.00	10.00	10.00
Barley grain (CDC Austenson)	88.65	59.40	29.25	—
Hybrid rye grain (KWS Bono)	—	29.25	59.40	88.65
Limestone	1.25	1.25	1.25	1.25
Mineral^†^	0.10	0.10	0.10	0.10
Chemical composition, % DM^‡^				
DM	85.54 ± 0.33	86.11 ± 0.30	86.70 ± 0.31	87.27 ± 0.34
OM	94.74 ± 0.64	95.16 ± 0.45	95.59 ± 0.27	96.01 ± 0.21
CP	16.60 ± 0.87	15.32 ± 0.57	14.01 ± 0.28	12.73 ± 0.17
ADF	10.07 ± 0.39	8.60 ± 0.31	7.09 ± 0.25	5.62 ± 0.23
NDF	25.84 ± 2.22	25.79 ± 1.52	25.73 ± 2.08	25.67 ± 3.30
Starch	43.63 ± 2.50	47.13 ± 1.61	50.74 ± 0.98	54.24 ± 1.20
Ether extract	2.34 ± 0.46	2.20 ± 0.38	2.05 ± 0.36	1.91 ± 0.42
Ca	0.52 ± 0.01	0.52 ± 0.01	0.51 ± 0.01	0.51 ± 0.01
P	0.38 ± 0.01	0.35 ± 0.01	0.31 ± 0.01	0.27 ± 0.01

^*^The treatment diets contained hybrid rye grain at 0%, 29.24%, 59.36%, and 88.60% of dietary DM for FCON, FLOW, FMED, and FHIGH, respectively.

^†^Mineral contained: Ca: 4.00 %; fat: 1.00%; Mn: 120,000 mg/kg; Cu: 60,000 mg/kg; Zn: 180,000 mg/kg; I: 5,000 mg/kg; Co: 750 mg/kg; Se: 750 mg/kg; vitamin A: 25,200 IU; vitamin D: 2,520 IU; vitamin E: 158 IU; 33 mg/kg of monensin (Elanco Animal Health, Greenfield IN) on a DM basis.

^‡^Chemical composition is expressed as means ± SD (*n* = 4).

Steers were weighed every 3 weeks during the study and on 2 consecutive days at the end of the study prior to feeding. Live BW gain was calculated by regressing unshrunk BW against days on feed as in Study 2. On day 98 of the finishing phase, freshly defecated feces were collected from at least 6 steers/pen (250 g/steer). Fecal samples were used for determination of starch concentration as described in Study 2.

Upon reaching a population target end weight of 665 kg (unshrunk), all steers were transported to a federally inspected abattoir (Cargill Meat Solutions, High River, AB, Canada). Hot carcass weight (**HCW**) was measured and used to determine dressing percentage by dividing HCW by the average BW measured at the end of the study after correction (4%) for gut fill. Carcass-adjusted final BW was calculated as the HCW divided by the observed dressing percentage for the whole population. Carcasses were then split into halves and chilled for 28 h. A certified grader authorized by the Canadian Beef Grading Agency (Calgary, AB, Canada) provided yield and quality grades. In addition, a computer vision grading system (VBG 2,000 e + v Technology GmbH, Oranienburg, Germany) was used to assess back-fat thickness, rib eye area, and marbling score between the 12th and 13th ribs. Liver scores were evaluated using the methods of Brown and Lawrence (2010) adapted to three classifications. Livers without a visible abscess or scarring received a score of 0; while livers with one or two small abscesses, or up to two to four well-organized abscesses under 2.54 cm in diameter were classified as mild. Livers with one or more abscesses greater than 2.54 cm in diameter or inflammation of liver tissue surrounding the abscess were classified as severe.

### Grain Processing and Feeding Management

Throughout Study 2 and 3, HR grain and barley grain were dry-rolled using a roller mill (KalRob Machining Ltd., Picture Butte, AB, Canada) to a target processing index between 75% and 80%. The processing index was measured by dividing the volume weight of the processed sample by the volume weight of the original grain sample (Yang et al., 2000). The proportion of fines were determined using a Penn State Particle Size Separator (Nasco Education, Fort Atkinson, WI, USA) using the 1.18-mm sieve and the pan with a 500-mL sample. The weight of the material on the pan was recorded and expressed as a percent of the total sample weight to determine the percent fines. The processing index and percent fines were recorded each time a new batch of cereal grain was processed (Table 3).

**Table 3.** Chemical composition of barley grain (*Hordeum vulgare L*.; CDC Austenson) and hybrid rye grain (*Secale cereale L*.; KWS Bono) used in the diets for growing and finishing steers

Item	Ingredient	
	Barley grain	Hybrid rye grain
Chemical composition, % DM^*^		
DM, %	90.42 ± 0.94	92.71 ± 0.58
OM	96.96 ± 0.60	98.12 ± 0.26
CP	16.47 ± 0.74	12.57 ± 0.71
ADF	8.39 ± 0.41	3.27 ± 0.20
NDF	24.40 ± 2.13	22.76 ± 2.95
Starch	50.57 ± 2.93	60.41 ± 1.57
Ether extract	2.18 ± 0.48	1.57 ± 0.36
Ca	0.07 ± 0.005	0.05 ± 0.015
P	0.40 ± 0.01	0.28 ± 0.01
Processing index, %^†^	75.51 ± 2.83	79.96 ± 2.25
Fines, %^‡^	4.07 ± 2.07	6.77 ± 1.17
7-h in vitro starch digestion, %^∥^	63.57 ± 4.68	67.19 ± 2.55

^*^Chemical composition was analyzed after cleaning the HR grain to remove ergot contamination. Values are expressed as means ± SD (*n* = 7).

^†^Processing index was measured as the volume weight (g/L) after processing expressed as a percentage of the volume weight before processing (Beauchemin et al., 2001; *n* = 4).

^‡^The proportion of fines was measured by dividing the weight of the processed grain that passes through the 1.18 mm sieve of the Penn State Particle Size Separator by the weight of the original sample (Beauchemin et al., 2001; *n* = 4).

^∥^7-h in vitro starch digestibility was determined by Cumberland Valley Analytical Services according to the procedure described by Johnson et al. (2020).

All steers were fed once daily between 0900 and 1200 hours targeting trace amounts of residual feed in the bunk. Diets were mixed and delivered using a horizontal mixer mounted on a truck (Kuhn RC 260V feed mixer; Kuhn North America, Inc., Brodhead, WI, USA; 2017 Freightliner 114SD; Daimler Truck North America, LLC, Portland, OR, USA). Trace minerals, vitamins, and feed additives were delivered to the truck using a micro-machine (Micro-Weigh System; Micro Technologies Feedlot Solutions). All diets were designed to supply 33 mg/kg of sodium monensin (Rumensin Premix; Elanco Canada Ltd., Mississauga, ON, Canada).

Residual feed in the bunk was removed and weighed on a weekly basis and a subsample of refusals from each pen were dried in a forced-air oven at 55 °C until achieving a constant weight to determine DM concentration. The weekly pen DMI was determined based on the difference between the amount of DM offered and the amount of DM refused. The DMI values were corrected for the number of steers in each pen and the number of days. Samples of all feed ingredients were collected twice per week, and all samples were dried in a forced-air oven at 55 °C until achieving a constant weight to determine DM. Dietary DM coefficients used in the feeding management software were modified on a weekly basis if the DM concentration of the fresh samples differed by more than two percentage units from the 3-week running average. Dried feed ingredient samples were composited by month (*n* = 3 in Study 2 and *n* = 4 in Study 3) on an equal DM weight basis. Dry samples were ground to pass through a 1-mm screen using a hammer mill (Christie-Norris Laboratory Mill, Christie-Norris Ltd, Chelmsford, UK). Subsequently, all ground feed samples were sent to Cumberland Valley Analytical Services (Waynesboro, PA, USA) for analysis of organic matter, CP, NDF, ADF, starch, ether extract, Ca, and P as described above. In addition, 7-h in vitro starch digestibility was determined by Cumberland Valley Analytical Services (Waynesboro, PA, USA) as described by Johnson et al. (2020). The dietary net energy values for maintenance (**NE**_**m**_) and gain (**NE**_**g**_) were estimated based on steer BW and growth as described by NASEM (2016). The retained energy for medium-framed steer calves was used (retained energy = [0.0635 × empty BW^0.75^] × empty BW gain^1.097^; NASEM 2016) using the mid-test BW adjusted to calculate the equivalent shrunk BW at 28% body fat. The maintenance energy requirement was calculated as 0.077 × equivalent shrunk BW^0.75^. The NE_m_ concentration in the diet was then calculated using the quadratic formula in Zinn et al. (2002) and the NE_g_ was calculated according to the study by Zinn and Shen (1998) using the equation: NE_g_ = NE_m_ × 0.877 to 0.41.

### Statistical Analysis

Crop production data were analyzed using the MIXED procedure of SAS (version 9.4, SAS Institute, Inc. Cary, NC) as a completely randomized design with fixed effects of cereal grain type and random effect of plot. Degrees of freedom were adjusted using the Kenward-Roger option.

Data for DMI, BW, ADG, and G:F were analyzed independently for Study 2 and 3 using the MIXED procedure of SAS (version 9.4, SAS Institute, Inc.) as a randomized complete block design with pen considered as the experimental unit and the random effect of block. For Study 3, the previous treatment (treatment in Study 2) and interaction between Study 2 and 3 treatments were tested. There were no interactions, so the previous treatment (Study 2) was included as a random effect, and the treatment applied (Study 3) was considered as a fixed effect. The degrees of freedom were adjusted using the Kenward-Roger option. Least square means were presented, and polynomial contrasts were used to evaluate whether linear, quadratic (Study 2), and cubic (Study 3 only) responses were present. Data pertaining to HCW, dressing percentage, rib eye area, back fat thickness, and marbling score were also analyzed using the model described above.

Categorical data (yield grade, quality grade, and liver abscess scores) were analyzed using the GLIMMIX procedure of SAS with a binominal error structure and logit data transformation. The model included the fixed effects of treatment and the random effects of pen, block, and the treatment imposed during Study 3. Means and standard errors of the mean (**SEM**) were reverse transformed for tabular presentation. Probability values reported are from analyses of transformed data while means and the SEM are from reverse-transformed data. For all analyses, results were declared significant when *P* ≤ 0.05 and trends were discussed when 0.05 < *P* ≤ 0.10.

## Results

### Study 1: Crop Production

The grain yield and density of HR were greater than that of barley (*P *= 0.04 and *P* < 0.01, respectively; Table 4). Likewise, the DM and starch concentrations of HR grain were greater than those of barley grain (*P* < 0.01). In contrast, the CP and NDF concentrations were less for HR than for barley grain (*P* < 0.01). Ether extract and Ca concentrations did not differ. Phosphorus concentration was greater for barley than that of HR (*P* < 0.01). When factoring yield and chemical composition, the DM and starch yields were greater (*P *= 0.03 and *P* = 0.04, respectively) for HR grain when compared with barley grain.

**Table 4.** Yield and chemical composition of barley grain (*Hordeum vulgare L*.; CDC Austenson) and hybrid rye grain (*Secale cereale L*.; KWS Bono) immediately after harvest

	Treatment^*^			
Item	Barley grain	Hybrid rye grain	SEM	*P* value
*n*	3	3		
Grain yield, kg wet/ha	3,558	4,667	265.4	0.04
Grain yield, kg DM/ha	3,160	4,231	237.9	0.03
Grain density, g/500 mL	285.1	366.1	4.44	<0.01
Chemical composition, % DM				
DM, %	88.8	90.7	0.23	<0.01
CP	16.2	11.9	0.46	<0.01
NDF	21.1	15.9	1.19	<0.01
Starch	54.8	63.8	0.81	<0.01
Ether extract	2.13	2.33	0.189	0.50
Ca	0.06	0.05	0.002	0.12
P	0.41	0.29	0.009	<0.01
Yield, kg/ha				
DM	3,160	4,231	237.9	0.03
CP	507.1	502.0	25.53	0.90
ADF	236.3	170.7	9.86	0.009
NDF	664.3	673.2	61.29	0.92
Starch	54.8	75.5	4.76	0.04

^*^Chemical composition was analyzed prior to cleaning the HR grain for ergot alkaloids. The values are expressed as means ± SD.

The straw yield (*P* = 0.06; Table 5) and DM yield (*P* = 0.08) tended to be greater for barley than for HR. Barley straw CP concentration (*P* = 0.01) and CP yield (*P* < 0.01) were greater for barley than for HR straw. While ADF concentration and the yield (*P* > 0.10) did not differ among barley or HR, the concentration of aNDFom (*P* = 0.03) and lignin (*P* = 0.047) of barley straw were lower than HR straw. In addition, the P concentration tended to be higher (*P* = 0.09) for barley straw than HR.

**Table 5.** Yield and chemical composition of barley straw (*Hordeum vulgare L*.; CDC Austenson) and hybrid rye straw (*Secale cereale L*.; KWS Bono) immediately after harvest

	Treatment			
Item	Barley straw	Hybrid rye straw	SEM	*P* value
*n*	3	3		
Straw yield, kg wet/ha	2,845	2,316	143.2	0.06
Straw yield, kg DM/ha	2,551	2,109	136.2	0.08
DM, %	89.6	91.1	0.74	0.24
CP	7.53	4.60	0.478	0.01
ADF	48.8	49.4	0.98	0.719
NDF	73.4	75.6	0.79	0.117
aNDFom	71.6	74.7	0.65	0.028
Lignin	7.08	7.71	0.158	0.047
Starch	0.53	0.50	0.062	0.73
Ether extract	0.68	0.75	0.096	0.60
Ca	0.34	0.34	0.018	0.99
P	0.09	0.07	0.007	0.09
Yield, kg/ha				
DM	2,551	2,109	136.2	0.08
CP	191.2	96.9	10.37	<0.01
ADF	1,250.2	1,041.7	90.54	0.18
NDF	1,871.8	1,595.9	99.72	0.12
aNDFom	1,826.9	1,576.1	101.1	0.15

### Study 2: Effect of Replacing Barley Grain With HR Grain for Growing Steers

Initial BW, final BW, DMI, and G:F were not affected (*P* ≥ 0.11; Table 6) by HR grain inclusion. However, a quadratic response (*P *= 0.02) was detected for ADG with a maximum point at BMED. The calculated NE_m_ and NE_g_, based on feed intake and growth, were not affected by HR grain inclusion (*P* ≥ 0.19). Fecal starch values were not affected and were < 1% of DM (*P ≥* 0.06; data not shown).

**Table 6.** Effect of replacing dry-rolled barley grain with dry-rolled hybrid rye grain on growth performance of steers during growing period (15 steers per pen with 8 pens per treatment)

	Hybrid rye inclusion^*^			SEM^†^	*P*-value		
Item	BCON	BMED	BHIGH		Treatment	Linear	Quadratic
Initial BW, kg	349	348	348	6.6	0.91	0.67	0.93
Final BW, kg	464	468	467	7.6	0.30	0.32	0.23
ADG, kg/d	1.84	1.94	1.90	0.027	0.03	0.12	0.02
DMI, kg/d	9.65	9.81	9.56	0.233	0.58	0.72	0.33
G:F, kg/kg	0.19	0.20	0.20	0.004	0.18	0.11	0.37
NEm, Mcal/kg^‡^	1.85	1.87	1.90	0.032	0.41	0.19	0.80
NEg, Mcal/kg^‡^	1.22	1.24	1.26	0.028	0.43	0.21	0.87

^*^The treatment diets contained hybrid rye grain at 0%, 29.24%, 59.36%, or 88.60% of dietary DM for FCON, FLOW, FMED, and FHIGH, respectively.

^†^Greatest SEM was reported.

^‡^Net energy values were calculated based on animal performance for the growing period as described by NASEM 2016.

### Study 3: Effect of Replacing Barley Grain With HR Grain for Finishing Steers

While initial BW was not affected (*P* ≥ 0.27; Table 7) by HR inclusion, shrunk final BW and carcass-adjusted final BW decreased quadratically with increasing HR inclusion (*P *≤ 0.02) such that the magnitude difference for BW, relative to FCON, was greater as HR inclusion increased. DMI linearly decreased with increasing HR inclusion (*P* < 0.01). Steer ADG and carcass-adjusted ADG responded quadratically (*P* ≤ 0.02), increasing from FCON to FLOW and decreasing thereafter. HR inclusion did not affect G:F or carcass-adjusted G:F (*P* ≥ 0.20). In addition, NE_m_ (*P* = 0.02) and NE_g_ (*P* = 0.02) linearly increased with increasing HR inclusion during the finishing phase. Fecal starch values were not affected by treatment and were less than 2% of dietary DM (*P ≥* 0.18; data not shown).

**Table 7.** Effect of replacing dry-rolled barley grain with dry-rolled hybrid rye grain on growth performance of steers during finishing period (15 steers per pen with 6 pens per treatment)

	Hybrid rye inclusion^*^				SEM^†^	*P*-value			
Item	FCON	FLOW	FMED	FHIGH		Treatment	Linear	Quadratic	Cubic
Initial BW, kg	466	466	467	467	10.8	0.52	0.27	0.90	0.32
Shrunk final BW, kg^‡^	647	647	639	622	12.5	<0.01	<0.01	0.01	0.99
DMI, kg/d	11.6	11.5	10.9	10.3	0.21	<0.01	<0.01	0.11	0.49
ADG, kg/d^∥^	1.69	1.74	1.63	1.51	0.028	<0.01	<0.01	<0.01	0.23
G:F, kg/kg^$^	0.145	0.148	0.152	0.147	0.0032	0.50	0.56	0.20	0.56
Carcass-adjusted final BW^¶^, kg	645	645	641	625	11.3	<0.01	<0.01	0.02	0.57
Carcass-adjusted ADG^¶^, kg/d	1.52	1.52	1.47	1.35	0.024	<0.01	<0.01	0.02	0.76
Carcass-adjusted G:F^¶^, kg/kg	0.131	0.132	0.135	0.130	0.0029	0.50	0.87	0.23	0.35
NEm, Mcal/kg^**^	1.70	1.75	1.76	1.77	0.021	0.10	0.02	0.36	0.71
NEg, Mcal/kg^**^	1.08	1.12	1.13	1.14	0.019	0.10	0.02	0.31	0.71

^*^The treatment diets contained hybrid rye grain at 0%, 29.24%, 59.36%, or 88.60% of dietary DM for FCON, FLOW, FMED, and FHIGH, respectively.

^†^Greatest SEM was reported.

^‡^Shrunk BW was calculated by multiplying final BW by 0.96 (NASEM, 2016).

^∥^ADG was determined by regressing BW on the day of the experiment.

^$^G:F was calculated for each pen as ADG divided by DMI.

^¶^Carcass-adjusted final BW was calculated as the hot carcass weight divided by the average dressing percentage of 59.9%, and then used to calculate ADG and G:F.

^**^Net energy values were calculated based on animal performance for the finishing period as described by NASEM (2016).

HCW decreased at an increasing rate with increasing inclusion of HR grain (quadratic, *P* = 0.02; Table 8). Increasing the proportion of HR grain in the diets linearly decreased back fat thickness (*P* = 0.04) and yield score (*P* = 0.03), but dressing percentage increased linearly (*P* = 0.02). There was no dietary effect on the rib eye area (*P* = 0.74). The proportions of carcasses classified as yield grade 2 quadratically increased (*P* < 0.01) with an initial decrease from FCON to FLOW followed by an increase with increasing HR inclusion. Increasing HR inclusion resulted in the opposite change in the proportion of carcasses classified as yield grade 3 (quadratic; *P *< 0.01) relative to yield grade 2. There was no effect of HR inclusion on the proportion of yield grades 1 and 4. The proportions of carcasses grading AAA (*P *< 0.01) and AA (*P *< 0.01) responded cubically where AAA was greater for greatest for FCON and least for FHIGH. Proportional changes in AA carcasses also responded cubically with the least values for FCON while values for FLOW and FHIGH were greater than FMED. The proportions of Prime and B4 were not affected by dietary treatments. The proportion of livers without abscesses were greatest for FCON intermediate for FLOW and FMED, and least for FHIGH (cubic, *P *< 0.01). Increasing the inclusion of HR (cubic, *P *< 0.01) increased the proportion of severe liver abscesses where values were greater for any inclusion of HR than FCON.

**Table 8.** Effect of replacing dry-rolled barley grain with dry-rolled hybrid rye grain on carcass characteristics of steers during finishing period (15 steers per pen with 6 pens per treatment)

	Hybrid rye inclusion^*^				SEM^†^	*P*-value			
Item	FCON	FLOW	FMED	FHIGH		Treatment	Linear	Quadratic	Cubic
HCW, kg	386	386	384	374	6.8	<0.01	<0.01	0.02	0.64
Dressing, %	59.71	59.67	60.05	60.19	0.204	0.09	0.02	0.58	0.36
Back fat, cm	1.63	1.67	1.57	1.45	0.072	0.11	0.04	0.22	0.68
Rib eye area, cm^†^	89.65	89.18	88.90	90.73	1.242	0.74	0.60	0.37	0.73
Yield score	3.47	3.52	3.41	3.12	0.123	0.07	0.03	0.12	0.96
Yield grade, %^‡^									
CBGA 1	5.63	3.33	6.15	6.98	0.921	0.07	0.10	0.08	0.07
CBGA 2	51.19	46.67	54.06	72.22	1.985	<0.01	<0.01	<0.01	0.96
CBGA 3	37.54	42.22	38.69	20.80	1.906	<0.01	<0.01	<0.01	0.32
CBGA 4	5.64	7.78	1.11	0.00	0.614	<0.01	0.97	0.97	0.98
Marbling, %	3.52	3.57	3.47	3.18	0.122	0.08	0.03	0.13	0.98
Marbling score^∥^	434.1	430.9	438.2	421.8	9.80	0.68	0.51	0.51	0.44
Quality grade, %^‡^									
CBGA Prime	0.00	1.11	1.11	0.00	0.214	1.00	1.00	0.97	1.00
CBGA AAA	78.89	70.00	72.78	66.19	1.821	<0.01	<0.01	0.40	0.02
CBGA AA	18.89	28.89	23.81	31.59	1.771	<0.01	<0.01	0.39	<0.01
CBGA B4	2.22	0.00	2.30	2.22	0.454	1.00	0.97	0.97	0.97
Liver score, %^$^									
Clear	70.00	44.45	49.60	32.70	1.964	<0.01	<0.01	0.045	<0.01
Minor	18.89	20.00	15.72	33.73	1.662	<0.01	<0.01	<0.01	<0.01
Severe	11.11	35.56	34.68	33.57	1.777	<0.01	<0.01	<0.01	<0.01

^*^The treatment diets contained hybrid rye grain at 0%, 29.24%, 59.36%, or 88.60% of dietary DM for FCON, FLOW, FMED, and FHIGH, respectively.

^†^Greatest SEM was reported.

^‡^Percentage of total according to Canadian Beef Grading Agency (CBGA; Calgary, AB, Canada).

^∥^According to U.S. Department of Agriculture (USDA) where 200 to 299 = trace; 300 to 399 = slight; 400 to 499 = small; 500 to 599 = modest; and 600 to 699 = moderate.

^$^Liver scores were classified as clear, minor, or severe as adapted from Brown and Lawrence (2010).

## Discussion

### Crop Production

Winter cereals provide an opportunity for producers to diversify their cropping strategy altering timing of seeding, spraying, and harvest (Clark, 2008; Larsen et al., 2018; Rusche et al., 2020) but there is a paucity of data directly comparing yield and chemical composition of HR and barley grain. When compared among winter cereals, HR has been reported to be among the greatest yielding, exceeding conventional fall rye, triticale, and wheat by 35%, 48%, and 23%, respectively (KWS, 2023). In this study, HR grain yield was 31.2% greater than barley grain when reported on a kg DM/ha basis. We are unaware of other studies comparing the yield of barley and HR. Similar to previously reported results (Krieg et al., 2017), we observed that CP and NDF concentrations were greater for barley than HR, but starch concentration was less resulting in lesser starch yield for barley than HR. Given the importance of starch for growing and finishing cattle, these data suggest that HR may provide an opportunity to increase starch yield, at least relative to barley grain.

While the HR variety grown was developed to have reduced ergot risk (Hansen et al., 2004), ergot contamination occurred in the present study. In fact, the total ergot alkaloid concentrations in HR were 5.33 ± 2.51 mg/kg with ergocristine/inine, ergocryptine/inine, ergotamine/inine, ergocornine/inine, ergometrine/inine, and ergosine/inine making up 42.33%, 17.86%, 14.54%, 12.34%, 7.84%, and 5.09% of the ergot alkaloids, respectively. As such, HR required cleaning to reduce ergot alkaloid concentration resulting in a final concentration of 0.29 ± 0.09 mg/kg. While not the focus of this research, ergot alkaloids have been reported to decrease feed intake, growth, and feed efficiency in livestock (Klotz, 2015). Cleaning also appeared to affect the nutrient composition of the HR with numeric increases for CP and NDF, but a reduction in starch and ether extract relative to collection at harvest. Cleaning may have removed small weed seeds in addition to ergot-contaminated grain resulting in changes in chemical composition.

The straw yield was greater for barley than HR, and the barley straw had greater CP but lesser aNDFom and lignin concentrations. The CP concentration of barley straw in this study was well above the reported average (NASEM, 2016), likely due to drought conditions (Jensen et al., 2003) with means of 7.5% ± 0.92% CP reported. When considering yield, barley straw resulted in a greater yield of CP, while aNDF and lignin yield did not differ from HR. When calculated as a percentage of total yield, the HR grain represented 66.8% of the DM yield while barley grain represented 55.6% of the DM yield. We are not aware of data comparing straw yields for spring-seeded cereals and HR.

### Growing Phase

Given the rapid rate of ruminal fermentation for HR (Krieg et al., 2017; Pereira et al., 2022), we hypothesized that HR grain could be used as a partial replacement for barley grain in diets for growing cattle, but that increasing the dietary inclusion rate would decrease DMI and growth for finishing cattle resulting in negative effects on carcass characteristics and the severity of liver abscesses. Partially supporting our hypothesis, increasing the inclusion rate of HR did not affect DMI but quadratically affected ADG during the growing phase with the greatest ADG occurring when the blend of barley and HR were fed. Other studies have also reported beneficial effects of blended grain sources (Kreikemeier et al., 1987; Huck et al., 1998); however, positive associative effects are generally observed when cereal grains differ in rates of degradation or the site and extent of digestion (Axe et al., 1987). HR and barley grain are both rapidly degraded in the rumen, but the rate of degradation in the present study based on starch reactivity and 7-h in situ degradation, along with previous research (Krieg et al., 2017), is interpreted to suggest that rates and extents of digestion are greater for HR than barley grain, despite being processed to a lesser severity. However, in growing diets, grain accounted for a maximum of 60.22% of the diet and as such, the moderate starch concentrations and potential differences in starch availability did not appear to negatively affect responses until HR was included as the sole grain source. It should be noted that as HR inclusion increased, dietary starch concentration also increased. As such, it is impossible to separate effects of starch availability and starch concentration. Therefore, the quadratic response with increasing HR grain inclusion and, greatest ADG for BMED relative to other treatments, may be due to a slight increase in rapidly degradable starch supply or the slight increase in starch concentration. Further increases in HR inclusion may have led to a reduction in ruminal pH thereby compromising fiber digestibility (Calsamiglia et al., 2002; Yang and Beauchemin, 2007) and energy supply driving the reduction in ADG when HR was the sole cereal grain source. However, we did not measure ruminal pH or fiber digestibility during this study and, as such, further research is needed to confirm this speculation.

### Finishing Phase

When fed to finishing cattle, increasing inclusion of HR linearly reduced DMI, and quadratically affected final BW, ADG, and carcass-adjusted final BW and ADG without affecting G:F. The effects of increasing HR inclusion in this study are consistent with those reported by Rusche et al. (2020). Given the lack of effect on G:F, it is likely that the reductions for final BW, ADG, as well as carcass-adjusted values were caused by the reduction in DMI. Rusche et al. (2020) reported that increasing the proportion of HR grain at the expense of dry-rolled corn for finishing steers linearly decreased DMI, ADG, G:F, and carcass-adjusted final BW, and speculated that the responses may be related to greater risk for ruminal acidosis. In this study, increasing HR inclusion also increased starch content from 43.63% for FCON to 54.24% for FHIGH (DM basis). When combined with the greater rates of fermentation previously reported for rye than barley (Krieg et al., 2017) and based on laboratory analysis from the present study, it could be expected that increasing HR inclusion increased the supply of ruminally degraded starch. Large amounts of rapidly degradable starch result in the rapid production of large quantities of organic acids in the rumen decreasing ruminal pH and may lead to ruminal acidosis (Aschenbach et al., 2011; Schwaiger et al., 2013). Cattle exposed to ruminal acidosis have been reported to decrease DMI in response to low ruminal pH as a potential mechanism to prevent further increases in the ruminally degradable carbohydrate supply (Owens et al., 1998; Schwartzkopf-Genswein et al., 2004; González et al., 2012). While ruminal pH was not assessed, we did observe that HR inclusion during the finishing phase, at any inclusion rate, increased the risk for severe liver abscesses, supporting greater risk and severity of ruminal acidosis (Koenig et al., 2020; Lawrence, 2020). As such, part of the reduction in DMI with HR inclusion may be due to reduced ruminal pH.

In addition to potential effects on ruminal fermentation, it is possible that the presence of ergot alkaloids may be partially responsible for the reduction in DMI. As noted, the HR grain in the present study was contaminated with ergot alkaloids and even after cleaning, total ergot alkaloid concentrations were 0.29 ± 0.09 mg/kg. While it is expected that the concentration of ergot alkaloids after cleaning was sufficiently low enough to mitigate risk for effects on DMI, ergot alkaloids have been reported to reduce feed intake and animal performance at concentrations as low as 0.100 mg/kg (Coufal-Majewski et al., 2016), particularly when cattle are fed for relatively long periods (Klotz, 2015; Sarich et al., 2023). Based on analysis of the grain sources, increasing the inclusion of HR grain would be expected to increase dietary total ergot alkaloid concentration from 0 mg/kg (FCON) to 0.254 mg/kg (FHIGH). Nevertheless, we cannot exclude ergot alkaloids as a possible reason that HR reduced DMI in Study 3.

We observed that increasing the inclusion of HR grain in finishing diets quadratically affected ADG and final BW where partial replacement of barley grain (29.24% of DM) resulted in the greatest response. Combining cereal grain sources varying in ruminal fermentability has been reported to improve starch utilization and feed efficiency (Kreikemeier et al., 1987) as previously discussed for the growing phase. However, starch from barley and HR are both expected to have a rapid rate and high extent of ruminal digestion (Benninghoff et al., 2015; Humer and Zebeli, 2017), questioning why such a response may have occurred, particularly when diets contain high starch concentrations. That said, further increases in HR inclusion or the complete replacement of barley grain with HR reduced ADG, final BW, and G:F. As discussed above, the slower gain and reduced G:F are likely driven by the reduction in DMI observed with increasing HR grain at the expense of barley grain.

Increasing the proportion of HR grain in the diet quadratically reduced HCW such that increasing HR to 67% and 100% of the dietary cereal grain inclusion reduced HCW. In addition, there was a linear reduction in fat thickness, yield score, and general reductions in marbling and quality grades indicative of reduced intramuscular fat deposition. As discussed above, the reduction in DMI likely reduced energy intake thereby delaying carcass gain and fat deposition (Owens et al., 1995). Others have reported that HR inclusion reduced final BW, dressing percentage, HCW, and rib eye area for steers as HR inclusion rates increased at the expense of dry-rolled corn (Rusche et al., 2020). That said, in that same study, carcass fatness indicators such as rib fat, retail yield, USDA yield grades, and kidney, pelvic, and heart fat were not affected by dietary HR inclusion (Rusche et al., 2020). Interestingly, in Study 3, yield score and yield grade did not follow similar patterns despite evaluating similar characteristics albeit using differing methodologies. A certified grader provided assessment of carcasses and the yield and quality grades using visual observation; while yield score and marbling percentage were determined using the vision grading system. It is unclear why these systems differ in the nature of the response for the yield score and yield grade for this study; however, it may be possible that changes in rib eye area coupled with changes in intramuscular fat make it more difficult for human-based grading to capture differences.

Overall, the proportion of cattle with minor and severe liver abscesses displayed a cubic response with increasing inclusion of HR as a replacement for barley grain such that any diet that contained HR were greater than FCON. The prevalence of liver abscesses in feedlot cattle has been reported to range between 12% and 32% (Brink et al., 1990) but can be as high as 95% (Nagaraja and Lechtenberg, 2007). The combined proportions of cattle with minor and severe liver abscesses were 30.0%, 55.6%, 50.4%, and 67.3% for FCON, FLOW, FMED, and FHIGH, respectively. Cattle with severe liver abscesses have been reported to have lesser DMI, reduced growth, and diminished carcass yield and quality (Nagaraja and Lechtenberg, 2007; Brown and Lawrence, 2010; Reinhardt and Hubbert, 2015); all responses observed in this study as HR increased. However, we cannot attribute the performance responses directly to liver abscesses as there are several other contributing factors described above. That said, cereal grain type and grain processing are two factors influencing the prevalence and severity of liver abscesses (Nagaraja and Lechtenberg, 2007) such that diets high in rapidly fermentable starch during the finishing phase are thought to increase risk for development of the ruminal-acidosis ruminitis-liver abscess complex (Lawrence, 2020; Reinbold, 2020). Although ruminal pH was not measured in these studies, the decreasing DMI with increasing proportion of HR grain and the concomitant decrease in ADG, carcass weight, fat thickness, and marbling are likely indicators of ruminal acidosis supporting increased severity of liver abscesses.

## Conclusions

When comparing hybrid fall rye and spring barley in this study, HR had greater DM yield and starch concentrations relative to barley. Replacing 50% of the barley grain with HR increased ADG during the growing phase, but had no effect on final BW or DMI. In contrast, increasing HR inclusion at the expense of barley grain in finishing diets decreased DMI and growth performance of finishing steers suggesting that HR inclusion rates should not exceed 33% of the cereal grain in barley-based finishing diets. Although ruminal pH was not measured in Study 2 or 3, decreased HCW, back fat thickness, and carcass characteristics, and increased prevalence of severe liver abscesses with increasing HR inclusion are interpreted to indicate that replacement of barley grain with HR in finishing diets has the potential to increase risk for ruminal acidosis.

## Supplementary Material

txae059_suppl_Supplementary_Table_S1
