# Supplementary material for: Production and use of dry-rolled hybrid rye grain as a replacement for barley grain on growth performance and carcass quality of feedlot steers
Source: Transl Anim Sci. 2024 Apr 11;8:txae059. doi: 10.1093/tas/txae059 (PMC11067788; doi:10.1093/tas/txae059)
Supplement: txae059_suppl_Supplementary_Table_S1 [file txae059_suppl_supplementary_table_s1.docx]

Supplementary Table 1. Ingredient composition of transition diets fed to steers to transition from growing diets to their respective finishing diets over 20 d, each step was 5 d in duration with the final finishing diets fed on day 21.

|  | Stage of transition | | | | |
| --- | --- | --- | --- | --- | --- |
| Ingredient, % DM | Step 1 | Step 2 | Step 3 | Step 4 | Final |
| FCON |  |  |  |  |  |
| Barley silage | 38.40 | 28.60 | 20.60 | 14.60 | 10.00 |
| Barley grain | 60.22 | 70.00 | 78.00 | 84.00 | 88.60 |
| Hybrid rye grain | - | - | - | - | - |
| Limestone | 1.25 | 1.25 | 1.25 | 1.25 | 1.25 |
| Mineral^2^ | 0.10 | 0.10 | 0.10 | 0.10 | 0.10 |
| FLOW |  |  |  |  |  |
| Barley silage | 38.40 | 28.60 | 20.60 | 14.60 | 10.00 |
| Barley grain | 40.35 | 46.90 | 52.26 | 56.28 | 59.36 |
| Hybrid rye grain | 19.87 | 23.10 | 25.74 | 27.72 | 29.24 |
| Limestone | 1.25 | 1.25 | 1.25 | 1.25 | 1.25 |
| Mineral^2^ | 0.10 | 0.10 | 0.10 | 0.10 | 0.10 |
| FMED |  |  |  |  |  |
| Barley silage | 38.40 | 28.60 | 20.60 | 14.60 | 10.00 |
| Barley grain | 19.87 | 23.10 | 25.74 | 27.72 | 29.24 |
| Hybrid rye grain | 40.35 | 46.9 | 52.26 | 56.28 | 59.36 |
| Limestone | 1.25 | 1.25 | 1.25 | 1.25 | 1.25 |
| Mineral^2^ | 0.10 | 0.10 | 0.10 | 0.10 | 0.10 |
| FHIGH |  |  |  |  |  |
| Barley silage | 38.40 | 28.60 | 20.60 | 14.60 | 10.00 |
| Barley grain | - | - | - | - | - |
| Hybrid rye grain | 60.22 | 70.00 | 78.00 | 84.00 | 88.60 |
| Limestone | 1.25 | 1.25 | 1.25 | 1.25 | 1.25 |
| Mineral^2^ | 0.10 | 0.10 | 0.10 | 0.10 | 0.10 |

^1^The treatments diets contained hybrid rye grain at 0%, 29.24%, 59.36%, and 88.60% of dietary DM for FCON, FLOW, FMED, and FHIGH, respectively.

^2^Mineral contained: Ca: 4.00 %; fat: 1.00%; Mn: 120,000 mg/kg; Cu: 60,000 mg/kg; Zn: 180,000 mg/kg; I: 5,000 mg/kg; Co: 750 mg/kg; Se: 750 mg/kg; Vitamin A: 25,200 IU; Vitamin D: 2,520 IU; Vitamin E: 158 IU; 33 mg/kg of monensin (Elanco Animal Health, Greenfield IN) on a DM basis.
